# A Prospective, Randomized, Double-Blind, Parallel-Group, Placebo-Controlled Study Evaluating Meniscal Healing, Clinical Outcomes, and Safety in Patients Undergoing Meniscal Repair of Unstable, Complete Vertical Meniscal Tears (Bucket Handle) Augmented with Platelet-Rich Plasma

**DOI:** 10.1155/2018/9315815

**Published:** 2018-03-11

**Authors:** Rafal Kaminski, Krzysztof Kulinski, Katarzyna Kozar-Kaminska, Monika Wielgus, Maciej Langner, Marcin K. Wasko, Jacek Kowalczewski, Stanislaw Pomianowski

**Affiliations:** ^1^Department of Musculoskeletal Trauma Surgery and Orthopaedics, Postgraduate Center for Medical Education, Professor A. Gruca Teaching Hospital, Konarskiego 13, 05-400 Otwock, Poland; ^2^Department of Medical Biology, Laboratory of Immunology, The Cardinal Stefan Wyszynski Institute of Cardiology, Alpejska 42, 04-628 Warsaw, Poland; ^3^Department of Anesthesiology, Postgraduate Center for Medical Education, Professor A. Gruca Teaching Hospital, Konarskiego 13, 05-400 Otwock, Poland; ^4^Department of Radiology, Postgraduate Center for Medical Education, Professor A. Gruca Teaching Hospital, Konarskiego 13, 05-400 Otwock, Poland; ^5^Department of Orthopaedics and Rheumoortopaedics, Postgraduate Center for Medical Education, Professor A. Gruca Teaching Hospital, Konarskiego 13, 05-400 Otwock, Poland

## Abstract

**Objective:**

The present study aimed to investigate the effectiveness and safety of platelet-rich plasma (PRP) application in arthroscopic repair of complete vertical tear of meniscus located in the red-white zone.

**Methods:**

This single center, prospective, randomized, double-blind, placebo-controlled, parallel-arm study included 37 patients with complete vertical meniscus tears. Patients received an intrarepair site injection of either PRP or sterile 0.9% saline during an index arthroscopy. The primary endpoint was the rate of meniscus healing in the two groups. The secondary endpoints were changes in the International Knee Documentation Committee (IKDC) score, Knee Injury and Osteoarthritis Outcome Score (KOOS), Western Ontario and McMaster Universities Osteoarthritis Index (WOMAC), and analog scale (VAS) in the two groups at 42 months.

**Results:**

After 18 weeks, the meniscus healing rate was significantly higher in the PRP-treated group than in the control group (85% versus 47%, *P* = 0.048). Functional outcomes were significantly better 42 months after treatment than at baseline in both groups. The IKDC score, WOMAC, and KOOS were significantly better in the PRP-treated group than in the control group. No adverse events were reported during the study period.

**Conclusions:**

The findings of this study indicate that PRP augmentation in meniscus repair results in improvements in both meniscus healing and functional outcome.

## 1. Introduction

Globally, almost 4 million arthroscopies for meniscus pathologies are performed annually [1]. In young, active patients without cartilage damage, arthroscopic meniscus repair with suture and meniscus replacement have shown beneficial effects in long-term studies [2]. Meniscus repair, rather than partial meniscectomy, is usually reserved for young patients (<35 years old), with strict indications [3]. Approximately 52–93% of meniscal repairs will heal (with overall failure rate of 23.1%) [4–6]. So far, many techniques, such as the use of fibrin glue, mechanical stimulation, gene therapy, and administration of growth factors, have been employed to improve the healing rate after meniscus repair [7].

Few clinical trials have provided evidence for the use of the fibrin clot technique, which is widely implemented [8, 9]. Additionally, there are only single studies presenting* in vitro* and* in vivo* evidence for the use of platelet-rich plasma (PRP) [10–12], with no data to support statement that PRP loaded in matrices may produce improvement in healing compared with cell-free implants. Current evidence suggests that PRP may not be as potent as previously believed [13]. One matter of importance is the lack of prior published randomized controlled studies assessing the effect of PRP on meniscal healing. To study this effect, we designed a prospective, randomized, double-blind, parallel-group, placebo-controlled study. The purpose of this study was to investigate the effectiveness and safety of PRP application in arthroscopic repair of complete vertical meniscal tears located in the red-white zone. We hypothesized that arthroscopic meniscus repair with PRP application would result in both an improved healing rate and better functional outcomes.

## 2. Materials and Methods

### 2.1. Trial Design and Informed Consent

This was a parallel-group, superiority trial with equal randomization. The study protocol was approved by an appropriate Institutional Review Board and was publicly accessible before enrollment of the first patient. We performed the study in accordance with the ethical standards outlined in the 1964 Declaration of Helsinki, and we report the results according to the 2010 CONSORT statement. The potential benefits and risks of PRP application, meniscus repair, and follow-up were explained to each study patient. All patients provided written informed consent for participation in this study, and no patient declined to participate. The protocol was registered under the number 36/PW/2011 at the local clinical trial database and is publicly accessible (http://www.cmkp.edu.pl).

### 2.2. Eligibility Criteria

**Figure 1:**
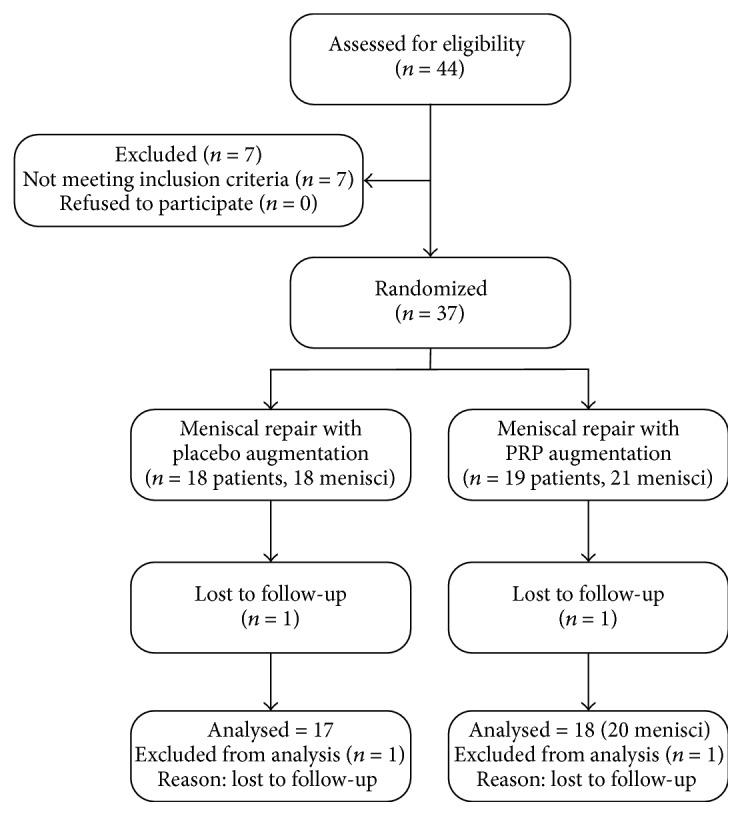
Flow diagram of the trial.

Patients were recruited from a single public knee clinic at a tertiary care, university health center between 2011 and 2013 (Figure 1). Thirty-seven patients with unstable complete vertical longitudinal tears in Cooper Zone 2 were enrolled. Of these 37 patients, 18 were randomized to undergo meniscus repair and receive placebo injection at the repair site (control group, 18 menisci repaired) and 19 were randomized to undergo meniscus repair and receive PRP injection at the repair site (PRP-treated group, 21 menisci repaired). Detailed inclusion and exclusion criteria are presented in Table 1. Patients had no other knee surgery before or after the follow-up period.

### 2.3. PRP and Thrombin Preparation

PRP and its activator (thrombin) were prepared using a protocol described previously by Everts et al. [14] and Mazzucco et al. [15]. Briefly, the PRP preparation procedure involved drawing 120 mL of venous blood before surgery and centrifuging the blood using a refrigerated centrifuge in a two-step process. The PRP solution was prepared by a single laboratory technician. The contents were validated using enzyme-linked immunosorbent assay (ELISA) and a blood analyzer [16–19]. The results of validation are presented in Online Supplementary Material 1. In the PRP-treated group, 8 mL of PRP solution was used, while, in the control group, 8 mL of sterile 0.9% saline was used. Autologous thrombin was obtained by recalcification of a patient's platelet poor plasma (PPP) with 120 mM calcium chlorate at a ratio 5 : 1. Thrombin was recovered after centrifugation and PRP/activator ratio 9 : 1 was used to induce gel formation.

This method allows for isolation of leukocyte- and platelet-rich plasma (L-PRP). It is found to be advantageous as it slowly releases growth factors over the period of about 7 days [20] and it supports the growth factors to act as an assembly of platelets and leukocytes in a complex fibrin matrix. So far leukocyte- and platelet-rich fibrin (L-PRF) has been evaluated in trauma surgery [21]. Unfortunately, as L-PRF cannot be used in the form of an injection we believe L-PRP offers an alternative method in delivering PRP into the meniscus.

### 2.4. Procedures

All operations were performed by the same senior orthopedic surgeon under spinal anesthesia. All menisci were repaired using standard procedures (rasping, reduction, and fixation). Fixation was performed via the all-inside technique using a FastFix device (Smith and Nephew, Cordova, TN, USA). In patients with a tear extending from the posterior horn to zone 2b (middle body) [22], additional sutures were placed via the outside-in technique using Prolene suture material (Ethicon, Somerville, NJ, USA) (Online Supplementary Material 2). Outside-in technique was used for middle body repair. All sutures were placed in an oblique manner with spacing every 5 mm.

PRP and placebo were prepared outside the operating room by a dedicated laboratory assistant in the BL2 facility. The preparations were then packed into sterile vials labeled with the patient ID and patient clinical study number according to a randomization list. PRP was activated using 20 mM CaCl_2_ (Teva, Basel, Israel) and 25 IU/mL autologous thrombin by the operative team. This double activation system was used to abolish the anticoagulative effect of the citrate present in the predonation blood bag [14]. It was then injected into the repair site of the meniscus with a double-chamber syringe. Needle localization was confirmed with arthroscopic visualization. Clot formation was observed around the repair site. No sutures were ruptured during injection. PRP or placebo was injected at the end of the procedure, under air arthroscopy. No drainage was applied to the operated knee joint. After discharge, patients were referred to outpatient physiotherapy units and encouraged to follow a unified rehabilitation protocol. In short, exercises with an increasing passive range of motion from 0 to 90 degrees in weeks 0–6 and full range of motion in weeks 6–9 were encouraged. Weight bearing as tolerated was allowed from postoperative day 1. All patients wore a hinged knee brace for 8 weeks, and the brace was locked in full extension for walking (during full weight bearing) for the first 6 weeks postoperatively.

### 2.5. Outcomes

The primary outcome was meniscus healing. It was assessed using second-look arthroscopy or 1.5 T magnetic resonance imaging (MRI) with a dedicated knee coil (Siemens, Erlangen, Germany). Those patients with a second-look arthroscopy had no MR assessment. Meniscus healing was assessed by two independent attending knee surgeons (arthroscopic surgery only) and two independent radiology consultants (MRI only), who were blinded to the patient allocation. We did not notice any intraobserver bias. The meniscal healing assessment was performed as described by Tenuta and Arciero [23], Online Supplementary Material 3. Complete healing was considered when full meniscus integrity was noted during the second-look arthroscopy or MRI. Partial healing was considered when there was at least 50% healing of the tear width and stable repair. Healing failure was considered when there was no visible healing or there was healing of less than 50% of the tear width or an unstable repair.

The secondary outcomes included pain assessment with the visual analog scale (VAS) and functional outcome assessment with the Knee Injury and Osteoarthritis Outcome Score (KOOS), Western Ontario and McMaster Universities Osteoarthritis Index (WOMAC), and International Knee Documentation Committee Subjective Knee Evaluation (IKDC) [24–26]. All secondary outcomes were assessed preoperatively, at 6 and 12 weeks postoperatively, and at 6, 12, 18, 24, 30, 36, and 42 months postoperatively. Patients were closely monitored for perioperative or postoperative complications. There were no changes to the protocol during the study duration.

### 2.6. Randomization

The randomization list for allocating patients to the study groups was generated using the “simple randomization” function on the Statsoft GraphPad QuickCalcs Website (http://www.graphpad.com/quickcalcs) [27]. We used sequentially numbered, opaque, sealed envelopes to conceal the allocation. Patients were consecutively enrolled and assigned to the study groups. Intervention assignment was performed after the start of surgery.

### 2.7. Blinding

The patients, the data collectors, and the assessors were blinded to the intervention type.

### 2.8. Statistical Analysis

We used the *R* statistical package (https://www.r-project.org/) for statistical analyses [28]. Differences in meniscus healing rates were assessed through analysis of a contingency table using Fisher's exact test. Odds ratios were calculated with 95% confidence intervals (CIs). All categorical data were analyzed using Fisher's exact test. The VAS score, KOOS, WOMAC, and IKDC score were analyzed using the two-tailed Mann–Whitney *U* test or unpaired *t*-test (after assessment for parametric or nonparametric distribution using the Shapiro-Wilk test) [29]. Results were considered statistically significant at a *P* value < 0.05. Sample size was calculated for the primary outcome (meniscus healing) according to the method described by Altman [30], with a two-tailed significance level at alpha = 0.05 and beta = 0.8, assuming a difference in the meniscus healing rate of 35% between the study groups based on previous studies [5, 31, 32]. Minimum recruitment level was estimated to be 16 patients per group. Assuming an attrition or noncompliance rate of 10% during the study, we aimed to recruit at least 18 patients per group.

## 3. Results

The study flowchart is presented in Figure 1. Follow-up ended on March 1, 2017. The median follow-up duration was 54 months (range, 45–69 months). Two patients were lost to follow-up for the primary outcome (1 in the control group and 1 in the PRP-treated group). All patients ware functionally assessed at 42 months after surgery. There were no significant differences in baseline characteristics between the groups (Table 2).

### 3.1. Primary Outcome

**Figure 2:**
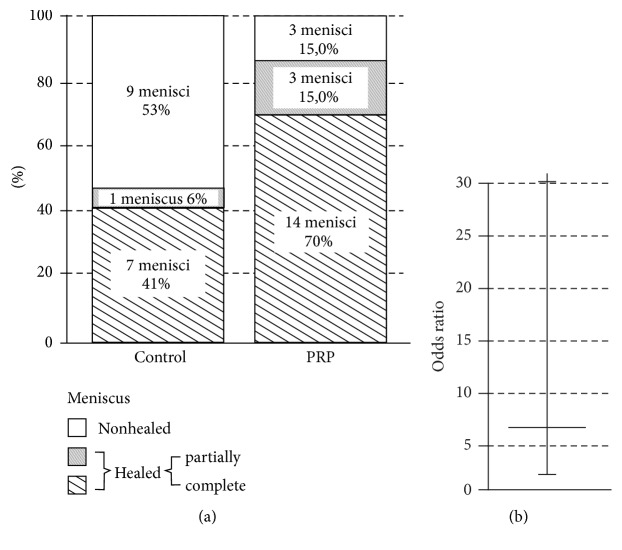
Meniscus healing rates. (a) Diagram showing the meniscus healing rate after meniscus repair with or without PRP. (b) Odds ratio for PRP augmentation. PRP, platelet-rich plasma.

Assessment of meniscus healing was performed at week 18 (±SD - 9 weeks) in both groups (Table 3). The healing rate of meniscus tears was superior in the PRP-treated group than in the control group (85% versus 47%, *P* = 0.048). The odds ratio for PRP-augmented meniscus healing was 6.375 (95% CI 1.35–30.14, *P* = 0.019) (Figure 2). We did not detect any significant influence of patient age or time from injury at the time of repair on the meniscal healing ratio. We believe this is due to the low number of patients in the study, as it was already proven that these factors influence meniscal repair outcome [6]. All patients with unhealed menisci underwent subsequent partial meniscectomies within 42 months of observation. All patients were included in secondary outcome assessments at 42 months (17 patients in the control group and 18 in the PRP-treated group).

### 3.2. Secondary Outcomes: Pain

The baseline pain characteristics (VAS and KOOS-pain) of the patients did not significantly differ between the control and PRP-treated groups. All patients undergoing meniscus repair (17 patients in the control group and 18 in the PRP-treated group) showed an improvement in pain scores (Tables 2 and 4). In the control group, the VAS score improved from 5.06 ± 0.13 (95% CI 4–6.11) before repair to 0.89 ± 0.08 (0.33–1.44) after repair (*P* < 0.001) and the KOOS-pain improved from 55.15 ± 1.04 (95% CI 46.49–63.81) before repair to 92.85 ± 0.43 (89.83–95.87) after repair (*P* < 0.001). In the PRP-treated group, the VAS score improved from 6.21 ± 0.13 (95% CI 5.13–7.29) before repair to 0.84 ± 0.10 (0.04–1.65) after repair (*P* < 0.001) and the KOOS-pain score improved from 58.81 ± 0.83 (95% CI 51.68–65.94) before repair to 96.06 ± 0.23 (94.22–97.91) after repair (*P* < 0.001). There were no significant differences in VAS score but KOOS-pain differed significantly (*P* = 0.035) between the control and PRP-treated groups at 42 months after repair. In a subgroup analysis, patients with healed menisci showed significant improvement in pain scores (VAS, KOOS-pain) over patients with nonhealed menisci.

### 3.3. Secondary Outcomes: Function

Functional outcomes were measured using the IKDC subjective scale, WOMAC, and the KOOS subscales (symptoms, function in daily living [ADL], sport/recreation, and knee related quality of life [QOL]). Each parameter improved over time in both groups. In the PRP-treated group, knee function was better at 42 months after repair than before repair. We noted that the IKDC score WOMAC score and KOOS (symptoms, ADL, sport/recreation, and QOL) were significantly better in the PRP-treated group than in the control group (Table 4). In a subgroup analysis, patients with healed menisci showed significant improvement in all functional scores over patients with nonhealed menisci.

### 3.4. Complications

No perioperative or postoperative complications were noted among patients who participated in the final follow-up. No increased risk of infection was observed in our study.

## 4. Discussion

Meniscus healing has always been a major challenge for orthopedic surgeons. All types of meniscectomies can lead to an increase in the risk of osteoarthritis [33]. Clinical studies comparing total and partial meniscectomy have documented the beneficial effects of meniscus preservation [34]. However only limited data exist and it so far fails to unequivocally support the benefits of meniscal repair over the partial meniscectomy [34–36]. Although reoperation rate for partial meniscectomy is significantly lower than for the meniscal repair (3% versus 20%), recent studies provided some evidence concerning the benefits of the latter. In the long-term follow-up (10 year) 78% of the patients who underwent the meniscal repair have no radiologic signs of osteoarthritis versus only 63% in the partial meniscectomy group [34]. So, the current practice is to preserve meniscus tissue, with minimal resection.

The most important finding of this study is that PRP augmentation improved the healing rate of complete vertical meniscus tears located in the red-white zone. Additionally, the functional outcomes at 42 months were better in patients treated with PRP-augmented meniscus repair than in those treated with only meniscus repair; however, pain levels were comparable between these patient groups.

The proposed mechanism of action of PRP in repaired menisci is twofold as follows: (a) it creates a scaffold for migrating cells and (b) it supplies the injury site with a range of growth factors, such as platelet-derived growth factor, vascular endothelial growth factor, and transforming growth factor beta 1. These growth factors are known to promote chemotaxis, angiogenesis, collagen matrix synthesis, and cell proliferation [37].

Limited data is available in animal studies of meniscal healing in a rabbit meniscal tear model. There were no differences noted in scar formation and filling in the defects [38] when tears were located in the vascular zone of the meniscus. Interestingly, similar results were obtained with cell-free collagen matrices in avascular zones of meniscus. Intriguingly, matrices loaded additionally with mesenchymal stem cells produced improved healing quality [12]. Ishida et al. showed positive results* in vitro* and* in vivo* while treating avascular meniscal defects with platelet-rich plasma; however, the meniscal defects size was different in the study by Zellner et al. [10, 12]. To our knowledge, there are only two human studies on PRP use in meniscus repair. Pujol et al. performed a case-control study on 34 patients who underwent open meniscus repair for horizontal meniscus tears. The authors found that clinical outcomes and healing rates were better with the introduction of PRP into the lesion at the end of the surgery than with standard open meniscus repair [39]. This previous study was focused on chronic horizontal tears and was neither blinded nor randomized. Therefore, the findings can be considered to provide an initial indication of the effectiveness of PRP use in meniscus repair. Griffin et al. performed a retrospective chart review, with revision surgery and the Lysholm score as the main outcome at a minimum of 2 years of follow-up. The authors failed to show any benefit of PRP augmentation; however, their study was underpowered for all the outcomes [40]. Additionally, they did not use any imaging modality for follow-up of the asymptomatic patients. Moreover, there were discrepancies in the tear characteristics between the groups. Furthermore, surgeon characteristics and demographics were inconsistent between the treatment groups.

Current studies observe a meniscal repair failure rate within 7–48% [4, 5]. These analyses do not usually evaluate the exact tear location, that is, distance from the synovial junction, red-red or red-white zone, and tear chronicity. Our failure rate was similar to that observed in previous and current studies for bucket-handle tears, older than six weeks and occurring more than 3 mm from the synovial junction [6, 23, 41–43]. For isolated meniscal repair Espejo-Reina et al. obtained a healing rate for complete vertical tears of 35% [44]. A low number of patients undergoing isolated meniscal repair was a weak point of the study.

Multiple factors can influence meniscal healing. Unfortunately, we did not monitor patient smoking status, as it was shown in recent studies to affect meniscal healing [45]. All our patients had concomitant ACL deficiency, which was shown to worsen meniscal healing. Last but not least, there was significant usage of oblique meniscal sutures for meniscal fixation. Also, current biomechanical studies show lower pull-up strengths of oblique versus vertical meniscal suture [46]. We believe all of those factors have influenced the high rates of failure observed.

The results of experimental studies support the hypothesis that PRP may improve healing of the meniscus through activation of fibrochondrocytes present within the avascular region of the meniscus [11, 47]. The release of growth factors from platelets has been shown to be associated with the initiation of a healing cascade, with an increase in the synthesis of deoxyribonucleic acid and extracellular matrix components [10, 33]. However, a study in rabbits by Zellner et al. showed that PRP in combination with hyaluronan collagen composite matrix failed to significantly improve meniscus healing in the avascular zone within 3 months [13]. Therefore, it is believed that mesenchymal stem cells are necessary for repair of meniscus lesions in the avascular zone [12]. Additionally, in a recent article by Lee et al. on a rabbit model of circular defect, PRP treatment failed to help in the production of meniscus cartilage, and it instead accelerated fibrosis and increased the production of catabolic molecules [48]. This might be a very important finding, as human studies have shown that catabolic activity in meniscus tissue may help identify patients who are at risk for progression of osteoarthritis following partial meniscectomy [49]. However, it should be noted that findings from animal and* in vitro* studies cannot be directly extended to clinical practice, as such studies are intrinsically unable to mimic the* in vivo* action of mesenchymal stem cells, intraarticular hematoma, and neuroinflammatory modulation present in a living human [12].

The strengths of this study are its prospective, randomized, and blinded nature and the use of standard repair techniques by a single surgeon. Additionally, independent assessors were used for outcomes.

The present study has some limitations. The number of study patients was low. Additionally, some patients did not undergo a second-look arthroscopy owing to their unwillingness to undergo another surgical procedure. Moreover, data on the actual number of platelets delivered to the repair zone were lacking. Importantly, 1.5 T MRI scanners are not at a disadvantage in comparison to 3 T in differentiating meniscal lesions and, in fact, meta-analysis shows that the specificity of 3-T MRI is lower than that of 1.5 T MRI with regard to the diagnosis of lateral meniscal tears [50], but still MRI images of the repaired meniscus can be difficult to interpret [49]. Therefore the measurement of the primary outcome might have been influenced by factors that could affect MRI images and the interpretation of the images. Furthermore, the generalizability of the findings of this study is limited owing to the low number of patients and the strict inclusion criteria. Nonetheless, this study showed that PRP augmentation in meniscus repair could provide significant and clinically important benefits.

## 5. Conclusions

Our study is the first blinded, prospective, randomized, controlled trial on the role of PRP augmentation in meniscus repair. The findings of this study indicate that PRP augmentation in meniscus repair results in a significant improvement in the rate of meniscus healing. PRP was found to improve the chances of meniscus healing by over six times (odds ratio 6.375 - 95% CI 1.35–30.14). Additionally, the possibility of adverse events related to the use of PRP is low.

## Figures and Tables

**Table 1:** Inclusion and exclusion criteria.

Inclusion criteria	Exclusion criteria
(i) Patients aged 18–55 years (ii) Complete vertical longitudinal tear >10 mm in length on MRI (iii) Unstable peripheral tear (iv) Meniscus lesion in Cooper zone 2; more than 4 mm from the rim (v) Meniscus injury 1–18 months prior to surgery	(i) Arthritic changes (Kellgren-Lawrence scale > 2) (ii) Degeneration or presence of crystals in the meniscus (iii) Meniscus lesion in the Cooper zone 0-1; less than 4 mm from the rim (pure red zone) (iv) Injury over 18 months prior to surgery (v) Concomitant surgical procedures (microfracturing, ligament reconstruction, fracture fixation, and trephination) (vi) Inflammatory diseases (i.e., rheumatoid arthritis)

MRI, magnetic resonance imaging.

**Table 2:** Baseline characteristics of the study patients in the control and PRP-treated groups.

	Control group (*n* = 18)	PRP-treated group (*n* = 19)	*P* value
Age (years)	26 (19–44)	30 (18–43)	*P* = 0.32
Sex (M : F)	15 : 3	15 : 4	*P* = 1.0
Time from injury (months)	3 (1–10)	2 (1–8)	*P* = 0.17

Education (high school : college/university)	10 : 8	12 : 7	*P* = 0.74

VAS score	5.06 ± 0.13 (4–6.11)	6.21 ± 0.13 (5.13–7.29)	*P* = 0.14
IKDC score	41.7 ± 0.84 (34.7–48.69)	40.92 ± 0.92 (33.09–48.74)	*P* = 0.88
WOMAC	38.61 ± 1.19 (28.74−48.49)	32.26 ± 0.90 (24.55–39.97)	*P* = *0.25*
KOOS			
(i) Pain	55.15 ± 1.04 (46.49–63.81)	58.81 ± 0.83 (51.68–65.94)	*P* = 0.53
(ii) Symptoms	44.84 ± 1.13 (35.44–54.25)	51.88 ± 1.15 (42.08–61.68)	*P* = 0.32
(iii) ADL	58.95 ± 1.34 (47.81–70.09)	66.68 ± 0.95 (58.55–74.81)	*P* = 0.28
(iv) Sport/recreation	24.44 ± 1.73 (10.03–38.86)	25.53 ± 1.32 (14.29–36.76)	*P* = 0.91
(v) QOL	22.57 ± 0.91 (15.02–30.12)	27.19 ± 0.76 (20.68–33.71)	*P* = 0.37

Data are presented as median (range) or mean ± standard error (95% confidence interval) unless otherwise indicated; PRP, platelet-rich plasma; VAS, visual analog scale; IKDC, International Knee Documentation Committee; WOMAC, Western Ontario and McMaster Universities Osteoarthritis Index; KOOS, Knee Injury and Osteoarthritis Outcome Score; ADL, activities of daily living; QOL, quality of life.

**Table 3:** Primary outcome assessment at 18 weeks (healing assessed using cumulative outcome, second-look arthroscopy, and MRI).

Cumulative outcome (assessed using MRI and second-look arthroscopy) (*P* = 0.048)		

Outcome	PRP-treated group (*n* of menisci)	Control group (*n* of menisci)
Healed	14	7
Partially healed	3	1
Failed	3	9

Second-look arthroscopy (*P* = 0.003)		

Outcome	PRP-treated group (*n* of menisci)	Control group (*n* of menisci)
Healed	11	3
Partially healed	2	1
Failed	1	8

MRI (*P* = 0.54)		

Outcome	PRP-treated group (*n* of menisci)	Control group (*n* of menisci)
Healed	3	4
Partially healed	1	0
Failed	2	1

MRI, magnetic resonance imaging; PRP, platelet-rich plasma.

**Table 4:** Secondary outcome assessment at 42 months (pain: VAS and KOOS-pain; function: IKDC, WOMAC, KOOS-symptom, KOOS-ADL, KOOS-sport/recreation, and KOOS-QOL).

	Control group	PRP-treated group	*P* value
VAS score	0.89 ± 0.08 (0.33–1.44)	0.84 ± 0.10 (0.04–1.65)	*P* = 0.15
IKDC score	84.77 ± 0.92 (78.24–91.29)	97.56 ± 0.63 (92.62–102.49)	*P* = 0.001
WOMAC	3.95 ± 0.33 (1.58−6.31)	0.95 ± 0.13 (−0.07–1.96)	*P* = *0.002*
KOOS score			
(i) Pain	92.85 ± 0.43 (89.83–95.87)	96.06 ± 0.23 (94.22–97.91)	*P* = 0.035
(ii) Symptoms	92.33 ± 0.48 (88.94–95.73)	96.23 ± 0.31 (93.79–98.67)	*P* = 0.029
(iii) ADL	95.14 ± 0.38 (92.47–97.81)	98.18 ± 0.13 (98.13–100.24)	*P* = 0.0004
(iv) Sport/recreation	77.65 ± 1.26 (68.73–86.56)	89.44 ± 0.86 (82.68–96.21)	*P* = 0.009
(v) QOL	66.18 ± 1.17 (57.94–74.42)	80.90 ± 1.09 (72.34–89.47)	*P* = 0.008

Data are presented as mean ± standard error (95% confidence interval); VAS, visual analog scale; KOOS, Knee Injury and Osteoarthritis Outcome Score; IKDC, International Knee Documentation Committee; WOMAC, Western Ontario and McMaster Universities Osteoarthritis Index; ADL, activities of daily living; QOL, quality of life; PRP, platelet-rich plasma.

## Supplementary Materials

Supplementary Materials Online Supplementary Material 1: characteristics of prepared PRP solution. Concentration of PLT in the PRP solution was roughly six times higher as compared to plasma samples. Online Supplementary Material 2: characteristics of patients in the study groups (e.g., the type of meniscus injured, number of sutures applied, and method of assessment). Online Supplementary Material 3: visual assessment of meniscal healing, healed, partially healed, and nonhealed menisci presented in arthroscopy (a second raw) and by magnetic resonance (a third raw).
