# Supplementary material for: A Prospective, Randomized, Double-Blind, Parallel-Group, Placebo-Controlled Study Evaluating Meniscal Healing, Clinical Outcomes, and Safety in Patients Undergoing Meniscal Repair of Unstable, Complete Vertical Meniscal Tears (Bucket Handle) Augmented with Platelet-Rich Plasma
Source: Biomed Res Int. 2018 Mar 11;2018:9315815. doi: 10.1155/2018/9315815 (PMC5866900; doi:10.1155/2018/9315815)

**Online Supplemetary Material**

Online Supplementary Material 1: Characteristics of PRP

|  | PRP | Normal serum | Citation |
| --- | --- | --- | --- |
| PLT count | 1980 (×1000/µL) | 150–400 (×1000/µL) |  |
|  |  |  |  |
| PDGF  VEGF  IGF  TGF-β1 | 410 ± 54 ng/mL  1430 ± 600 pg/mL  93 ± 29 ng/mL  91 ± 22 ng/mL | 8.5 ± 5.5 ng/mL  205 ± 121 ng/mL  80 – 300 ng/mL  0.05–0.9 ng/mL | [15,16]  [17,16]  [18]  [15] |

Data are presented as mean ± standard deviation or range.

PRP, platelet-rich plasma; PLT, platelet; PDGF, platelet-derived growth factor; VEGF, vascular endothelial growth factor; IGF, insulin-like growth factor; TGF, transforming growth factor

Online Supplementary Material 2: Additional characteristics of the patients in the study groups

| Patient no. | Group | Method of repair | No. of sutures | Cooper zone | Meniscus | Method of assessment |
| --- | --- | --- | --- | --- | --- | --- |
| 1 | Control | AI + OI | 4 + 2 | 2 | MML | SL |
| 2 | Control | AI | 2 | 2 | MM | SL |
| 3 | Control | AI | 4 | 2 | ML | MRI |
| 4 | Control | AI + OI | 6 | 2 | MM | SL |
| 5 | Control | AI | 2 | 2 | ML | MRI |
| 6 | Control | AI | 4 | 2 | MM | MRI |
| 7 | Control | AI | 2 | 2 | MM | MRI |
| 8 | Control | AI | 4 | 2 | ML | SL |
| 9 | Control | AI | 4 | 2 | MM | SL |
| 10 | Control | AI + OI | 6 | 2 | ML | MRI |
| 11 | Control | AI + OI | 8 | 2 | MM | SL |
| 12 | Control | AI | 6 | 2 | MM | SL |
| 13 | Control | AI | 4 | 2 | ML | SL |
| 14 | Control | AI | 4 | 2 | MM | SL |
| 15 | Control | AI | 4 | 2 | MM | SL |
| 16 | Control | AI | 4 | 2 | MM | SL |
| 17 | Control | AI | 2 | 2 | ML | SL |
| 18 | Control | AI + OI | 6 | 2 | MM | MRI |
|  |  |  |  |  |  |  |
| 19 | PRP | AI + OI | 6 | 2 | MM | MRI |
| 20 | PRP | AI | 2 | 2 | MM | SL |
| 21 | PRP | AI + OI | 6 | 2 | MM | MRI |
| 22 | PRP | AI | 4 | 2 | ML | SL |
| 23 | PRP | AI + OI | 6 | 2 | MM | SL |
| 24 | PRP | AI + OI | 6 | 2 | MM | SL |
| 25 | PRP | AI | 4 | 2 | MM | SL |
| 26 | PRP | AI + OI | 6 | 2 | MM | SL |
| 27 | PRP | AI + OI | 6 + 4 | 2 | MML | SL |
| 28 | PRP | AI | 4 | 2 | MM | SL |
| 29 | PRP | AI | 4 | 2 | MM | SL |
| 30 | PRP | AI + OI | 8 | 2 | MM | SL |
| 31 | PRP | AI | 4 | 2 | MM | SL |
| 32 | PRP | AI + OI | 6 | 2 | MM | SL |
| 33 | PRP | AI + OI | 6 | 2 | MM | MRI |
| 34 | PRP | AI | 4 | 2 | MM | MRI |
| 35 | PRP | AI | 6 | 2 | MM | MRI |
| 36 | PRP | AI + OI | 10 | 2 | MM | SL |
| 37 | PRP | AI | 2 + 2 | 2 | MML | SL |
| 1–18 *vs.* 19–37 |  | *P* = 0.22 | *P* = 0.13 | *P* = 0.98 | *P* = 0.09 | *P* = 0.61 |

AI, all-inside; OI, outside-in; SL, second-look arthroscopy; MM, medial meniscus; ML, lateral meniscus; MML, medial and lateral meniscus

Online Supplementary Material 3: Meniscal healing.


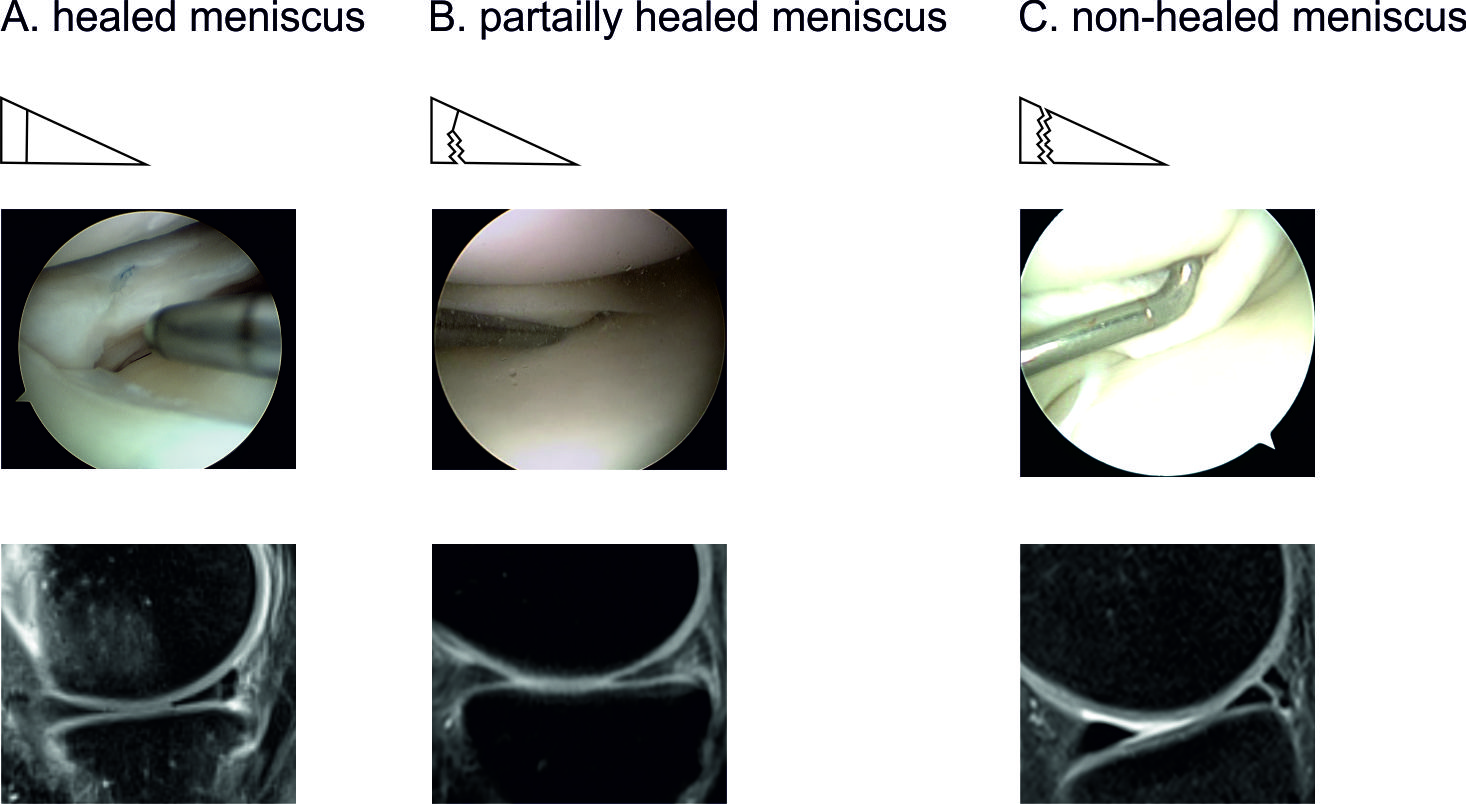

Supplement: Supplementary Materials — Online Supplementary Material 1: characteristics of prepared PRP solution. Concentration of PLT in the PRP solution was roughly six times higher as compared to plasma samples. Online Supplementary Material 2: characteristics of patients in the study groups (e.g., the type of meniscus injured, number of sutures applied, and method of assessment). Online Supplementary Material 3: visual assessment of meniscal healing, healed, partially healed, and nonhealed menisci presented in arthroscopy (a second raw) and by magnetic resonance (a third raw). [file 9315815.f1.docx]
